# Effect of timely initiation of breastfeeding on child health in Ghana

**DOI:** 10.1186/s13561-015-0044-8

**Published:** 2015-03-20

**Authors:** Rita Fosu-Brefo, Eric Arthur

**Affiliations:** 1 Department of Economics, University of Cape Coast, Cape Coast, Ghana; 2 Department of Economics and Statistics University of Benin, Benin City, Nigeria

**Keywords:** Breastfeeding, Timely Breastfeeding, Exclusive breastfeeding, Child health

## Abstract

**Background:**

Early initiation of breastfeeding and exclusive breastfeeding practices have been argued to be one of the important ways of ensuring child health. Unfortunately, owing to modernization, most nursing mothers fail to adhere to such practices. This is believed to be a factor contributory to poor child health in Ghana. Thus, this study investigated the effect of timely initiation of breastfeeding on child health in Ghana.

**Methods:**

Cross sectional data using secondary data based on the positivism approach to research was employed. The Ordinary least squares and the Instrumental variables approach were used in estimating the effect of breastfeeding and other socio demographic indicators on the health of the child. Data for the study was sourced from the 2008 round of the Ghana Demographic and Health Survey.

**Results:**

The results indicate that timely initiation of breastfeeding, both immediately and hours after birth are important factors that influence the child’s health. Additionally, factors such as the wealth of the household, mother’s education, age and size of the child at birth and age of the mother are important factors that also influence the health of the child in Ghana.

**Conclusion:**

The findings imply that efforts should be made on encouraging appropriate breastfeeding practices among nursing mothers to ensure proper child development and growth in Ghana.

## Background

The health of the child at the early stages of life is important for the proper growth of the child. It has been suggested that poor child health has adverse consequences on school attendance and performance and thus likely to affect human capital development of any nation. Given the importance of the health of the child, several attempts have been made to ensure the proper development of children in many contexts. International organisations such as the World Health Organisation (WHO), United Nations International Children Education Fund (UNICEF), and other related Non-Governmental Organisations have given much attention to child health. Ensuring an improved child health is important as it helps in reducing child mortality and influencing the future health outcomes of the child, in the areas of education and the ability to participate in the economy. Indeed, the Millennium Development Goals 1 and 4 aims to reduce child mortality and the prevalence of underweight in children under five years of age. This requires the identification of appropriate interventions that will help improve child health. One of such interventions has been argued to be timely initiation of breastfeeding.

Timely initiation of breastfeeding is defined as putting the new born to the breast either immediately or within an hour of birth. It is believed to contribute immensely to the sustainable health of the child by way of curbing malnutrition, gastroenteritis and strengthening the immune system of the child against diseases [1]. Timely initiation of breastfeeding, according to Setegn et al. [2] is not only the easiest, but also the most cost effective and most successful intervention in improving the health of the newborn. Given this identified relationship, health personnel have been on the campaign to create awareness and to encourage mothers to start breastfeeding their infants early. In addition to timely initiation of breastfeeding, exclusive breastfeeding and appropriate complementary feeding practices are also considered as essential elements for the satisfactory growth and development of infants and for prevention of childhood illness. Exclusive breastfeeding means that the infant is fed only breast milk for a period of six months with no other solids or liquids, including water, being given to the child, with the exception of drops or syrups consisting of vitamin or mineral supplements or medicines.

According to World Health [3], 22% of neonatal deaths could be prevented, if all infants are put to breast within the first hour of birth. It is not surprising therefore, that breastfeeding has become a major issue of concern to local and international healthcare organisations. UNICEF [4] reported that less than 40% of infants under 6 months of age were exclusively breastfed in 2009. This raises a concern for the development of the child and future prospects given the importance of feeding practices for the wellbeing of the child. The goals of Healthy People as at 2010 for breastfeeding were an initiation rate of 75% and continuation of breastfeeding of 50% at 6 months and 25% at 12 months postpartum [5]. Bellagio Child Survival Study Group highlighted breastfeeding as the lead prevention intervention to improve child health and survival and estimated that breastfeeding could prevent 13% of all deaths of children aged under-five years (about 1.3 million lives per year). The Collaborative Study Team of World Health Organisation also asserted that the risk of dying from infectious diseases throughout the first year of life was higher for non-breastfed infants. The study reported that in the first two months of life, non-breastfed infants had almost six-fold greater risk of dying from infectious diseases than breastfed infants. Given the stress of work and the challenges of occupation in the modern world, some women have resorted to bottle-feeding, hence, reducing the amount of time recommended for breastfeeding. This has important consequences on the health of children and their future participation in the economy.

In Ghana, there have been a number of programs and policies to promote and maintain child health. These programs include bed netting, vitamin A supplement, measles and polio vaccines, deworming exercise, exclusive breastfeeding and Oral Rehydration policies. Additionally, policies such as the adoption of the 1991 Baby-Friendly Hospital Initiative (BFHI) and the Ghana Breastfeeding Promotion Regulation 2000 (Legislative Instrument 1667) have been implemented with a focus of improving child health. Recently, the Government of Ghana adopted the High Impact Rapid Delivery (HIRD) approach as a national strategy to reduce child mortality. The HIRD approach bundles core health and nutrition interventions and delivers them in the heart of communities where families tend to lack access to healthcare facilities and lack even the most basic knowledge on how to manage common childhood disease. Despite the success of such interventions, there seems to be a lot to do in improving the health of the Ghanaian child, especially in encouraging mothers on the need to breastfeed their infants early and for an extended period. The GDHS [6] reported that progress has been slow in addressing chronic undernutrition or stunting in Ghana. The report indicated that the proportion of stunted children under 5 years of age decreased from 34% in 1988 to 31% in 1998 and rose again to 35% in 2003, before decreasing to 28% in 2008. This situation is undesirable and therefore needs to be addressed.

Under-five mortality in Ghana was high at 74.40% per 1000 live births in 2010 [7]. The causes of under-five mortality, according to Agble et al. [8], includes malaria (34%), neonatal causes (29%), pneumonia (15%), diarrhoea diseases (13%), measles (3%), AIDS (6%), and malnutrition (52%). The 52% attributed to malnutrition is high, accounting for more than half of under-five mortality in Ghana. The 2008 Ghana Demographic and Health Survey (GDHS) reported that the percentage of mothers who started breastfeeding their infants within one hour of birth had increased from 46% in 2003 to 52% in 2008, showing only an increase of 8% over the past 5 years. Additionally, the percentage of mothers who started breastfeeding their infants within the first day of birth increased from 75% to 82% in 5 years. Thus, the percentage of infants who were started on breast milk right after birth is less than the 75% envisaged by the goals of Healthy people. Thus, it becomes important to understand the effect of early initiation of breastfeeding on child health to inform mothers on the importance of breastfeeding for the proper growth of the infant in Ghana.

In the literature, some authors have studied the effect of breastfeeding on child health. In addition, socio-economic factors such as income, education, residence, labour force participation have been argued to affect child health, alongside breastfeeding. Unfortunately, most studies do not consider the timing of initiation, which is also important for the health of the child. In Ghana, Edmond et al. [9] who sought to assess the contribution of the timing of initiation of breastfeeding reported that out of a sample of 10,947 breastfed singleton infants born between July 2003 and June 2004, breastfeeding was initiated within the first day of birth in 71% with 70% who were exclusively breastfed during the neonatal period. Hong [10] on the effect of the duration of breastfeeding on child health reported that the duration of breastfeeding is an important factor that improves child health in Ghana. Annim [11] however reported in Ghana that the duration of breastfeeding was significant, but worsened child health for the period of his study. Tampah-Naah and Kumi-Kyereme [12] reported that mothers who perceived their infants to be average in size were more likely to practice exclusive breastfeeding. These few studies have been conducted in Ghana, but do not address the effect of timely initiation of breastfeeding on child health.

In addition to the importance of breastfeeding on the child's health, some social, economic and demographic factors of the mother can also affect the health of the child. These have been documented in previous studies. Frost et al. [13], Glewwe [14] and Webb and Block [15], Senauer and Garcia [16] and Mugo [17] are some of the studies that have documented the importance of social, economic and demographic factors on child health. Factors such as income, education, and modern attitudes about health care, birth order, maternal autonomy and mother’s labour force participation are some of the factors that have been identified in the literature. For instance, Mugo [17] reported that mother's labour force participation has a greater impact on the health of the child among the socio-economic factors investigated. Frost et al. [13] reported that maternal education was an important factor in the health of the child. This study therefore examines the effect of timely initiation of breastfeeding on child health in Ghana, whiles controlling for some of the socio-demographic factors of the mother/family.

## Methods

### Data

The study used cross sectional data from the 2008 round of the Ghana Demographic and Health Survey (GDHS). The GDHS is nationally representative with a large sample size. The data is collected by the Ghana Statistical Service, with technical support from the United States Agency for International Development (USAID) and Opinion Research Corporation (ORC Macro). It provides data on a wide range of monitoring and impact evaluation indicators in the areas of population, health and nutrition of women and children in developing countries. It is carried out once every five years. The strategic objective of the Measure DHS is to improve and institutionalise the collection and use of data on reproductive health by host countries for program monitoring and evaluation and for policy development decisions. The 2008 GDHS collected data on the health of children and women, in addition to the demographic profile of the households/individuals in the survey. We use a sample of 2449 infants born five years to the survey in the study. Following the World Health Organisation [18] growth standards measurement, the height for age z scores was used in this study as a measure of child health. The height for age z (HAZ) score is a measure of chronic malnutrition or stunting in children. The control variables in the study include wealth, education, employment, access to safe drinking water, gender of the child, the child’s age and the size of the child at birth.

### The model

This study is based on the Grossman [19] demand for health model. The main idea behind Grossman’s model is that individuals make informed choices over their life cycle to improve their health status by investing in health care and conforming to health enhancing behaviours and practices given their initial health status, which is assumed to be inherited from their parents and the environment. The individual is assumed a utility maximiser who faces a budget constraint that limits the amount of health care and other goods that he/she can consume. Children under the age of five depend mostly on their mothers/family for feeding and other essential needs. Hence, this study assumes that the mother derives utility from the good health of the child. The mother therefore engages in child health production using market and non-market or behavioural inputs [20]. The analysis in this study follows Strauss and Thomas [21] as outlined in Ajakaiye and Mwabu [22] by assuming that the decision of ensuring good health for the child is the responsibility of the mother. The mother’s utility function is specified in Equation 1:

1 $$ U=U\left(C,L,A,H,\xi \right) $$

In equation 1, the utility function of the mother (U) is specified as a function of the consumption of purchased goods (C), labour supply (L), the health of the child (H), Socio-demographic factors (A) and unobserved characteristics (*ξ)* such as heterogeneity in taste. The health of the child (H) is measured using the height for age z score. The health (H) of the child is specified in Equation 2 as:

2 $$ H=H\left(N,C,\mu \right) $$

In equation 2, H is specified as a function of child health inputs (N) such as timely initiation of breastfeeding. These inputs and behaviours are under the control of the mother. C comprises of child specific characteristics such as gender, age, etc. *μ* represents unobserved characteristics. The health production function incorporates behavioural choices and therefore not just a technical relationship.

The mother maximises the utility function subject to the budget constraint of income specified in Equation 3 as:

3 $$ {P}_cC+{P}_nNc=W $$

In equation 3, *P*_*c*_ is the price of consumption goods not related to the production of health, *P*_*n*_ is the price of purchased health inputs, *W* is the income of the household which is made up of both labour and non-labour income. Implicit in equation 3 is the time constraint comprising of time allocated to the production of health, leisure and labour supply, which is equal to total time endowment.

Further, by substituting equation 2 into equation 1 and maximising subject to the budget constraint in equation 3, we end up with equation 4, which presents the demand for child health by the mother, or in other words, a child health production function:

4 $$ H=H\left(Pn,Pc,A,W,\varepsilon \right) $$

In equation 4, *H* is a measure of child health status, which is a function of health input prices (*Pn),* price of consumption goods (Pc), socio-demographic status (A) and income (W). All of these characteristics are assumed exogenous to the individual’s decisions about their behaviours, investments, time allocation and resource allocation as far as their health is concerned. Unobserved factors, which influence demand for each of the health inputs and outputs, are captured by *ε,* which encompass not only tastes but also innate healthiness and other individual characteristics.

Equation (5) is just a functional form expressing the relationship between the health of the child and the other related variables. Thus, from equation 4, we specify equation 5 for the purpose of estimation as:

5 $$ \mathrm{H}=\upbeta \mathrm{N}+\uppi \mathrm{A}+\updelta \mathrm{C}+\upvarepsilon $$

N, A, and C in equation 5 represent child feeding practices (N), socio-demographic status of the mother/family (A) which includes wealth, education, etc. and child specific characteristics (C) respectively. β, π and δ are vector of parameters associated with N, A and C respectively. H is a measure of the health of the child, which is measured as height for age z score. We estimate equation 5 using both the ordinary least squares estimation technique and the instrumental variables technique.

A multivariate regression analysis was carried out in this paper. Previous studies have adopted one of three approaches, the logistic model, the ordinary least squares model and the instrumental variables approach. The analysis in this paper was carried out using the ordinary least squares estimation and the instrumental variables technique. We adopt the method of the ordinary least squares and the instrumental variables in this study due to the appropriateness of the two approaches. The dependent variable is a continuous variable. Some studies have categorised it enabling them to use the logistic regression model in their estimation. However, such re-categorisation leads to a loss of information. Since we have full information with the present measurement of the variable, this study considers it more appropriate to use the ordinary least squares regression approach. The instrumental variable technique additionally tests for endogeneity of the dependent variable. Some studies have suspected breastfeeding to be endogenous to child health. Thus, the use of the instrumental variable approach will help deal with such issues if present by using caesarean section as an instrument. The Wu-Hausman test for endogeneity was conducted to test whether the variable breastfeeding is endogenous or otherwise after the estimation of the regression model. The Breusch-Pagan/Cook-Weisberg test was used to test for the presence of heteroskedasticity in the model. Further, the Ramsey Regression Equation Specification Error Test [23] was carried out to test whether non-linear combinations of the fitted values help explain the response variable. Finally, the identification test was conducted for the instrumental variable model to find out whether the model was identified.

## Results

Tables 1 and 2 present a description of the dataset used in this study. Out of a sample of 2,449, the average z score was −1.055 with a standard deviation (SD) of 1.734 as reported in Table 1. Additionally, the maximum and minimum heights for age z scores were approximately plus and minus 6. Mothers on average breastfeed their babies for 16 weeks, with some mothers breastfeeding to the 58th week. The mean age for the child and the mother were recorded to be 29 weeks and 30 years respectively. The data also suggests that on average people spent about 18 minutes to get to the water source, with a minimum and maximum of zero (indicating having pipe borne water in household) and 120 minutes respectively.

**Table 1:** **Descriptive statistics of the variables**

**Variables**	**Mean**	**Standard deviation**	**Minimum**	**Maximum**
Height-for-Age	−1.055	1.734	−5.99	6
Duration of Breastfeeding	16.332	8.058	0	58
Child Age	28.644	17.230	0	59
Mother’s Age	30.191	6.997	15	49
Time to water source	17.347	19.572	0	120

Source: Author’s computation from the GDHS 2008*.*

**Table 2:** **Descriptive statistics of categorical variables**

**Variable**	**Frequencies**	**Percentages**	**Cumulative%**
**Mother’s Education**			
No education	930	38.1	38.1
Primary	569	23.31	61.41
Secondary	889	36.42	97.83
Tertiary	53	2.17	100
**Wealth of household**			
**Poor**	1,343	55.02	55.02
**Middle**	396	16.22	71.24
**Rich**	702	28.76	100
**Child Size**			
Small	345	14.28	14.28
Average	734	30.38	44.66
Large	1,337	55.34	100
**Sex of child**			
Female	1,213	49.69	49.69
Male	1,228	50.31	100
**Mother’s Occupation**			
Not Working	230	9.47	9.47
Informal Sector	2,136	87.98	97.44
Formal	62	2.55	100
**Initiation of breastfeeding**			
Immediately	963	39.94	39.94
Hours after birth	1,047	43.43	83.37
Days after birth	401	16.63	100

Source: Author’s computation from the GDHS 2008*.*

Furthermore, Table 2 suggests that majority of the mothers had no education (38.1%) with 36.42% and 2.17% having secondary and tertiary education respectively. 55.34% of the children were reported to have large size at birth with 30.38 having average size. 14.28% had small size at birth. Additionally, majority of the households (55.02%) were in the poor wealth quintile with 16.22% and 28.76% in the middle and rich wealth quintiles respectively. There was approximately an equal distribution of male and female children in the sample. Additionally, 43.43% of the mothers reported of starting their babies on breast milk hours after delivery, with 39.94% starting the babies on breast milk immediately. 16.64% started breastfeeding their babies days after delivery. Finally, with regard to mother’s occupation, the dataset suggests that majority of the women (87.98%) responded to be in the informal sector.

Table 3 presents the results from the regression model. Model one in Table 3 is the results from the Ordinary Least Squares (OLS) model and model two (IV) is the results from the Two Stage Least Squares (TSLS) model. The Wu-Hausman test for endogeneity rejected the endogeneity of timely initiation of breastfeeding in the model. The instrument used however passed the weak identification test from the results in Table 3. Thus, even though the results for both the Ordinary Least Squares and the instrumental variables have been presented, the focus of discussion will be on the Ordinary Least Squares model (OLS) results presented in Table 3 as model one (OLS). The post estimation test in the OLS results from column one indicates an R-squared (R^2^) of 13.3%, which implies that 13.3% of the variation in child health is explained by the explanatory variables used in this study. This value is quite low but according to Wooldridge [24], low R^2^ in regression equations is common especially for cross sectional analysis. Though R^2^ is an indication of the goodness of fit of the model, the size of the R^2^ is not as important as the statistical and economic significance of the independent variables [24]. In addition, the RESET test (with a p value of 0.3887) indicates that the model is correctly specified with no omitted variables. The test for multicollinearity indicates that the model is free from multicollinearity given that the mean variance inflation value of 1.51 is less than the critical value of 10. The results from the Breusch-Pagan/Cook-Weisberg test for heteroskedasticity however indicated the presence of heteroskedasticity; hence the robust standard errors were produced.

**Table 3:** **Results from the model**

**Explanatory variables**	**Model 1: OLS**	**Model 2: IV**
**Timely Breastfeeding**		0.107(0.268)
**Initiation of Breastfeeding (Days)**		
Breastfeeding immediately after birth	0.257***(0.0751)	
Breastfeeding hours after birth	0.158*(0.0952)	
**Duration of Breastfeeding**	−0.0428***(0.00536)	−0.0427***(0.00541)
**Male**	−0.0503(0.0676)	−0.0529(0.0674)
**Mother’s education (No education)**		
Primary	−0.0875(0.0928)	−0.0887(0.0929)
Secondary	0.0180(0.0872)	0.0242(0.0931)
Tertiary	0.490*(0.257)	0.501(0.312)
**Wealth (Poor)**		
Middle	0.131(0.0944)	0.136(0.103)
Rich	0.375***(0.0904)	0.369***(0.112)
**Child Size (Small)**		
Average	0.135(0.102)	0.145(0.110)
Large	0.291***(0.0945)	0.287***(0.0996)
**Child’s age**	−0.0179***(0.00246)	−0.0178***(0.00252)
**Mother’s age**	0.0185***(0.00504)	0.0178***(0.00514)
**Time to water source**	0.000908(0.00171)	0.000730(0.00187)
**Mother’s Occupation (not working)**		
Formal	0.0514(0.127)	0.0452(0.123)
Informal	−0.335(0.259)	−0.346(0.292)
Constant	−0.901***(0.218)	−0.929*(0.548)
Observations	2,312	2,311
R-squared	0.133	0.130
Ramsey Reset Test (p-value)	1.01(0.38)	3.80(0.14)
Mean Variance Inflation	1.52	1.65
Breusch-Pagan/Cook-Weisberg Test	52.86(0.00)	
Under Identification Test (p-value)		72.782(0.00)
Weak Identification Test (p-value)		74.628(0.10)
Wu-Hausman F test (p-value)		0.97908(0.10)

***p < 0.01, **p < 0.05, *p < 0.1.

Robust standard errors in parentheses.

Source: Authors own computation from GDHS 2008.

The results presented in Table 3 suggests that timely initiation of breastfeeding in model 2 (IV), had no effect on the health of the child. However, in model 1 (OLS), where two proxies for breastfeeding were used (breastfeeding immediately after birth and breastfeeding hours after birth), the results suggest that breastfeeding immediately after birth and breastfeeding hours after birth are both significant in influencing child health. Additionally, duration of breastfeeding had a significant effect on child health in both models. Thus, children who are breastfed immediately or hours after birth had a higher HAZ compared to those who are breastfed much later. The sign is positive and significant at the 5% level. Furthermore, the longer the breastfeeding period, the lower the HAZ score which is contrary to the expectation of the study. The results also indicate that the wealth of the household, the education of the mother and the age of the mother are important factors that influence the health of the child. Additionally, the size of the child at birth and the age of the child have significant effect on the health of the child. The results indicate that the age of the child has a negative effect on the health of the child, whiles the size of the child at birth has a positive effect on child health in Ghana. Additionally, mother’s age has a positive effect on the health of the child. The distance to the nearest water source and the occupation of the mother were not found to have any significant influence on the health of the child.

### Discussion

The description of the data suggests that some mothers did not breastfeed their infants, suggesting probably either breastfeeding the babies through the bottle, or using supplementary feeds for the child, which can have consequences for the health of the child and the child’s proper growth. Indeed this is characteristic of most developing countries. This mostly happens due to work pressure, as the mother may have to resume work since there might be no source of income to back her up in the period of nurturing the child, especially if the family is not an affluent one with some amount of resources to keep up. Indeed, this might explain why some mothers do not even breastfeed their infants, in the quest to return to work. In such situations, the babies are mostly left with the grandmother, a relative in the house, or probably older siblings to take care of.

The results from Table 3 indicate that timely initiation of breastfeeding measured as either immediate breastfeeding after birth or breastfeeding hours after birth are all significant in influencing child health. Breastfeeding immediately after birth and hours after birth are both positive and significant at the 5% and 10% levels respectively. The coefficient of breastfeeding immediately after birth is higher than the coefficient of breastfeeding hours after birth, suggesting that the contribution to child health is higher for mothers who breast-feed their infants immediately after birth compared to those who breast-feed their infants hours after birth. Additionally, the coefficient of breastfeeding hours after birth has a weakly significant effect on the health of the child as indicated from the results. This result implies that timely initiation of breastfeeding improves child health and aids the proper growth of the child. This finding is consistent with the World Health [3] recommendation of a positive effect of timely breastfeeding on child health. This result further supports previous studies by Edmond et al. [9], who also reported a positive effect of breastfeeding on child health. This thus suggests that as part of the initiatives in Ghana to promote child health, mothers should be educated on the importance of breastfeeding their infants immediately after birth, if possible.

We also find that duration of breastfeeding has a negative effect on the health of the child. The coefficient on the duration of breastfeeding is negative and statistically significant at 1%, contrary to our expectation. The negative effect is suspected to be due to mothers who feed their wards beyond the recommended time for exclusive breastfeeding. W.H.O recommends exclusively breastfeeding the infant for six months before introducing supplementary feeds, which is supposed to replace the breast milk after sometime. If this is not done, it might rather lead to a worsening effect on the health of the child, instead of the proposed positive benefits of exclusive breastfeeding. This finding is consistent with the results of Hong [10] and Annim [11] in Ghana. Hong [10] reported that longer duration of breastfeeding is associated with child stunting. Among the reasons alluded to by Hong [10] is a potential bi-causal relationship in which the mothers of malnourished children breastfeed for too long. Brakohiapa et al. [25] had also asserted a link between longer breastfeeding and child stunting earlier. The authors asserted that prolonged breastfeeding (more than 19 months) potentially reduces food intake and engenders malnutrition. Hence, even though mothers are encouraged to immediately and exclusively breastfeed their babies after birth, it is important to know when to introduce appropriate food supplements for the child to enhance the child’s health.

The wealth of the household is also statistically significant at 1%. This indicates that compared to poorer mothers/households, richer households are more likely to have healthier babies. This is not surprising as the wealth of the household indeed determines what can be put on the table for the mother, and thus affects the health of the child through the quality of the breast milk produced because of appropriate food intake of the mother. In addition, poorer mothers may be forced to end breastfeeding earlier than required to return to work, especially recalling that majority of the women in our sample were found to be in the informal sector. Thus, returning to work on time becomes important and this might affect the feeding practices of the mother, and that of the child who depends on the mother. In the context of Ghana, a developing country with high levels of poverty, it is not surprising that wealthier households have healthier babies.

Another factor that was found in this study to influence child health is the Mothers’ education and the age of the mother. The result for mother’s education (tertiary education) is positive and significant at the 5%. This means that mothers with tertiary education are more likely to have healthier babies compared to mothers with no education. Indeed, this confirms the predictions of the Grossman [19] model that education makes the individual more effective in the use of health inputs. Thus, educated mothers are able to assimilate information regarding the best practices to improve the health of their children, and are able to follow suit to improve the health and wellbeing of the babies than the uneducated. However, this benefit of education happens at the tertiary level. Even though, the coefficient for mothers with secondary education is positive, the result is not significant. This can be associated with the fact that a woman who has received higher education has knowledge of childcare practices, which helps her in the child upbringing. These findings are in conformity with works done by Annim [11] in Ghana. Thus, to promote child health in Ghana, it will be necessary to encourage women to pursue education, probably to the tertiary level as suggested by our findings. In addition, the results indicate that the age of the mother has a positive effect on the health of the child. This suggests that older mothers had healthier babies than younger mothers in this study. This might be due to the biological make-up of the woman, which probably may have an effect on the health of the child.

We also find that child characteristics, such as age of the child and size of the child at birth have significant effects on the health of the child. The age of the child is negatively associated with the child’s health while the size of the child at birth is positively associated with the child’s health, at the 1% level of significance. The positive and significant effect of the size of the child at birth on the health of the child has already been alluded to by some studies and conforms to the earlier finding by Annim [11] in Ghana. This positive relation is explained by the fact that average and larger sized babies’ might have a stronger immune system, which helps resist sickness and prevent anaemia. Thus, “smaller-sized” babies need to be given the needed attention to make sure they develop like their “normal” counterparts with average or large size at birth. Additionally, the significance of the age of the child suggests that as the child grows, health is likely to deteriorate, except when the child is given the needed attention and nutritional supplements to keep the child still healthy. Indeed, Grossman [19] predicts that health deteriorates with age and thus health investments are necessary in reducing the rate of health deterioration. Gyimah [26] posits that the growing up of children exposes them to dirt or other contaminated objects if considerable care and attention is not given, which can affect their health negatively. This notwithstanding, most children also become very active, coupled with the stress of school. Thus, it becomes important to give proper attention to the child as the child grows and seek appropriate care and hospital check-ups to ensure proper child growth.

## Conclusion

The study set out to investigate the effect of timely initiation of breastfeeding on child health in Ghana using the Grossman [19] demand for health model as a justification. The ordinary least squares and the instrumental variable techniques were used in analysing the data. The results indicate that breastfeeding is important in improving child health in Ghana. Particularly, timely initiation of breastfeeding, which was measured as starting the new born on breast milk either immediately after birth or hours after birth, were both significant in affecting child health. We find that the effect of starting the infant on breast milk immediately after birth is higher than starting the child on breast milk hours after birth. Additionally, the age of the child, size of the child at birth, wealth of the household, age of the mother and education are important in influencing the health of the child. Thus, we recommend that policy should aim at addressing these factors, particularly encouraging mothers and educating them on the importance of appropriate feeding practices for their infant and starting their infants on breast milk early to improve child health in Ghana. Mothers should also be educated to pay attention to their growing infants and seek health care appropriate care for the child to ensure the proper growth of the child.
